# An analysis of two island groups as potential sites for trials of transgenic mosquitoes for malaria control

**DOI:** 10.1111/eva.12056

**Published:** 2013-02-22

**Authors:** Clare D Marsden, Anthony Cornel, Yoosook Lee, Michelle R Sanford, Laura C Norris, Parker B Goodell, Catelyn C Nieman, Sarah Han, Amabelia Rodrigues, Joao Denis, Ahmed Ouledi, Gregory C Lanzaro

**Affiliations:** 1 Vector Genetics Laboratory, Department of Pathology, Microbiology, and Immunology, School of Veterinary Medicine, University of CaliforniaDavis, CA, USA; 2 Department of Entomology, University of CaliforniaDavis, CA, USA; 3 National Institute of Public Health (INASA)Bissau, Guinea-Bissau; 4 Université des Comores, rue de la CornicheMoroni, Grande Comore, Union of Comoros

**Keywords:** *Anopheles gambiae*, Bijagós, Comoros, genetically modified mosquitoes, island population, isolation

## Abstract

Considerable technological advances have been made towards the generation of genetically modified mosquitoes for vector control. In contrast, less progress has been made towards field evaluations of transformed mosquitoes which are critical for evaluating the success of, and hazards associated with, genetic modification. Oceanic islands have been highlighted as potentially the best locations for such trials. However, population genetic studies are necessary to verify isolation. Here, we used a panel of genetic markers to assess for evidence of genetic isolation of two oceanic island populations of the African malaria vector, *Anopheles gambiae* s.s. We found no evidence of isolation between the Bijagós archipelago and mainland Guinea-Bissau, despite separation by distances beyond the known dispersal capabilities of this taxon. Conversely, the Comoros Islands appear to be genetically isolated from the East African mainland, and thus represent a location worthy of further investigation for field trials. Based on assessments of gene flow within and between the Comoros islands, the island of Grande Comore was found to be genetically isolated from adjacent islands and also exhibited local population structure, indicating that it may be the most suitable site for trials with existing genetic modification technologies.

## Introduction

With 2009 marking the first field trials of genetically modified *Aedes aegypti* L. mosquitoes (Enserink 2010), and subsequent releases in 2010 and 2011 (Harris et al. 2011; Mumford 2012), it may appear that the era of transgenics for vector control has begun. However, the use of genetically modified mosquitoes (GMM) has been the subject of much debate and remains highly controversial (Enserink 2010; Ostera Gr 2011; Lehane and Aksoy 2012; Mumford 2012). This has created a need for thorough transparent scientific evaluation of the success of, and risks associated with, GMM releases prior to widespread deployment (Alphey et al. 2002). In particular, there has been concern over the potential for unexpected negative side effects associated with genetic modification (Alphey et al. 2002), which has led to calls to identify isolated field sites for GMM trials to minimize the potential for escapees (James 2005). Genetic tools are particularly appropriate for evaluating isolation because where populations become isolated, genetic differences arising from evolutionary processes such as mutation and genetic drift should accumulate, resulting in divergence from other populations (Hartl and Clark 2006). In contrast, movement of mosquitoes between contiguous populations should erode any potential genetic divergence, resulting in homogenized gene pools (Hartl and Clark 2006).

Human malaria is a parasitic infection spread exclusively by Anopheline mosquitoes which continues to result in approximately 655 000 deaths annually despite considerable investment in vector control strategies (WHO 2011). This persistent health burden, combined with the declining efficacy of traditional control methods due to physiological (Reimer et al. 2008) and behavioural resistance (e.g. early or outdoor feeding, Reddy et al. 2011), highlights the urgent need for new control approaches. In particular, attention has turned towards GMM technologies which aim to introduce transgenes into the mosquito population so that it is either suppressed (population suppression) or replaced with a variant that is unable to transmit disease (population replacement) (Coleman and Alphey 2004). These approaches may be either self-limiting, whereby GMM exhibit reduced fitness which results in transgenes disappearing from a population after releases are discontinued, or self-propagating, whereby genetic modifications include a gene drive system that enables transgenes to spread rapidly through wild populations and to be maintained at high frequencies between generations (Benedict and Robinson 2003; Coleman and Alphey 2004; Windbichler et al. 2011; Beech et al. 2012).

In order for GMM approaches to be implemented for large scale cost-effective malaria control, it will ultimately be necessary to utilize a self-propagating approach. However, due to their inherent design it would be very challenging to halt the spread of the transgene from a self-propagating GMM if negative side effects were observed following release into wild populations (Benedict and Robinson 2003). As such, it has been recommended that the first field trials of GMM in any species should be self-limiting so that the spread of the transgene can be halted by terminating releases (Benedict and Robinson 2003). In Anophelines, self-propagating technologies incorporating a gene-drive system linked to an appropriate transgene have not yet been developed, but considerable progress has been made towards modifying the Anopheline immune system to be refractory to the malaria parasite in the laboratory (i.e. malaria-resistant mosquitoes e.g. Corby-Harris et al. 2010; Dong et al. 2011; Isaacs et al. 2011). There is a now the need for suitable field trial sites to be identified so that the performance and behaviour of these self-limiting GMM and their associated transgenes can be assessed in wild populations. These sites need to be isolated, so that the risk of potential escapees is low (James 2005).

In sub-Saharan Africa, members of the *Anopheles gambiae s.l*. species complex (Giles) are the most significant vectors of human malaria. However, in many regions, the majority of malaria transmission is attributable to the highly anthropophilic species, *An. gambiae s.s*.. Genetic data has shown that *An. gambiae s.s*. is comprised of two incipient species, known as the M and S molecular forms. Where M and S co-occur in West and Central Africa they have been shown to exhibit strong reproductive isolation with rates of cross-matings typically <1% (Della Torre et al. 2001; Tripet et al. 2001), although it is noteworthy that higher rates of hybridization have been observed in the the most western part of the range (Oliveira et al. 2008; Caputo et al. 2011; Marsden et al. 2011). Elsewhere in Africa, only the S molecular form is found (Della Torre et al. 2001). Although strong reproductive barriers have been found between the molecular forms, within the molecular forms gene flow is extensive even between locations separated by several hundreds of kilometres (e.g. >1500 km S form, Slotman et al. 2007), despite the limited natural dispersal range of *An. gambiae* (<7 km with wind, Gillies and De Meillon 1968; Touré et al. 1998; Lounibos 2002). As such, oceanic islands have been identified as providing the best options for isolated field trial sites because large water bodies should pose significant barriers to movement of *An. gambiae* (Gillies and De Meillon 1968; Touré et al. 1998; Lounibos 2002). There are a relatively limited number of islands within the range of *An. gambiae* where: (i) *An. gambiae* is the primary vector of malaria and is responsible for active malaria transmission and (ii) where the island is located sufficiently far away from the mainland to be potentially isolated. Moreover, studies of both oceanic (Moreno et al. 2007; Marshall et al. 2008) and lacustrine islands (Chen et al. 2004; Kayondo et al. 2005) have demonstrated that human-assisted dispersal has the potential to connect geographically isolated populations of *An. gambiae* (Lounibos 2002). As such, it is important to verify the level of isolation between island and mainland sites. Given the potential for human-assisted dispersal, it is unlikely that an island exhibiting complete isolation exists. However, islands genetically distinct to, and exhibiting limited evidence of gene flow with, mainland sites, should pose lower risk. In this study, we assessed the level of connectivity between mainland Africa and two oceanic island groups, the Bijagós archipelago in West Africa and the Comoros Islands in East Africa, to evaluate their suitability as trial sites for GMM release.

## Methods

### Study site description

The Bijagós archipelago is composed of some 88 islands and islets situated just off the coast of Guinea-Bissau. These flat and low elevated islands collectively cover 900 km^2^ and support forest, savannah, floodplain, and mangrove habitats, as well as temporary and permanent agricultural areas. Malaria in the Bijagós islands is classed as hyperendemic (11–50% prevalence), with >100 malaria cases/1000 people (WHO 2011). The islands support a resident human population of ∼27 000, distributed across ∼20 of the islands, and we assessed three of these located 52-93 km from the mainland. Orango is the largest of the Bijagós islands (270 km^2^) and the furthest from the mainland. We also assessed the islands of Bubaque (85 km^2^) and Formosa (140 km^2^). These three islands have an isolation index of 10–17 according to the United Nations Environment Programme (UNEP) classification, which assesses the isolation of an island from potential colonization sources by calculating the sum of the square roots of the distances to the nearest equivalent or larger island, the nearest island group or archipelago and the nearest continent (UNEP 2010).

The Comoros Islands are a volcanic archipelago, located in the Indian Ocean, ∼700–800 km from the coast of Eastern Africa. Here, we assessed three of the four islands of the Union of Comoros, (Grande Comore, Moheli and Anjouan but not Mayotte), along with a site on mainland Tanzania, which is an important trading partner due to a free trade agreement between the two countries (Yssouf et al. 2011). The three islands each have an UNEP isolation index of 49 (UNEP 2010), however, they differ greatly in terms of size and topography. The largest island, Grande Comore totals 1148 km^2^, and has a resident population of ∼330 000. An active volcano, Karthala, reaching 2360 m is found in the Southern part of the island, and forms part of a belt of higher elevation (>500 m) that runs north to south. The highly permeable volcanic substrate results in an absence of surface water on the island. As such, *An. gambiae s.s*. larval sites are largely restricted to outdoor cisterns, which are widely used across the island to store rainwater for domestic use. In fact, according to Mouchet et al. (2008) malaria was only introduced to the island following the widespread construction of water cisterns in the 1920s. Moheli is the smallest island (∼290 km^2^), with a population of ∼40 000. The island is largely forested and consists of wide valleys with multiple rivers. The majority of Moheli is low elevation (<500 m), with the exception of a ridge reaching 600 m which dissects the centre of the island. The third island, Anjouan, is the most densely populated (∼280 000 people across 424 km^2^). The topography is severe, consisting of a number of steep ridges which reach up to 1500 m in elevation dissected by rivers and mountain streams. Across the islands, malaria is classified as mesoendemic (51–75% prevalence) to hyperendemic (11–50% prevalence). The number of malaria cases is highest on Grande Comore where most parts of the island report >100 cases/1000 population compared with 1–50/1000 on Moheli and Anjouan (WHO 2011).

### Anopheline sampling

On the Bijagós archipelago, mosquitoes were collected from a single site from each of the islands of Formosa, Bubaque and Orango, and from two sites on mainland Guinea-Bissau, in October to November of the 2009 rainy season (Table 1). On the Comoros, mosquitoes were collected from five to six sites from each of the islands of Grande Comore, Moheli and Anjouan, during the February 2011 rainy season (Table 1), and from the Ilala district in Dar es Salaam, Tanzania. At sites where adults were found indoors, mouth aspirators were used. However, at most sites in the Comoros, and islands of Bubaque and Orango in Guinea-Bissau, indoor resting adults were not found and so larvae were collected. Larval collections were made from pools of standing water, such as in roads, rice fields or swamps within or near villages. We specifically sampled specimens representing different larval stages, and made collections from multiple pools and/cisterns at each site, to limit over-sampling of relatives, which could inflate estimates of population differentiation.

**Table 1:** Sampling site description and overview of Anopheline species detected by PCR and morphological ID at each site

					*Anopheles* sp. by PCR				*Anopheles* sp. by morphology^+^			
						
Site (abbreviation)	Lat	Long	Elevation	Site description	AG *s.s*.	AM	AA	UNK	AG *s.l*.	AC	AP	UNK
*GUINEA-BISSAU*												
Mainland												
Antula (ANT)	11.91005	−15.58374	0	Roadside pool	124/127	3/127			X			
Prabis (PRA)	11.80066	−15.74332	8	Roadside pool	98/104	8/104			X			
Formosa												
Abu (ABU)	11.46144	−15.91411	0	Roadside pool	48/50	2/50			X			
Bubaque												
Bruce (BRU)	11.22319	−15.87378	0	Roadside pool	60/67	6/67	1/67		X			
Orango												
Eticoga (ETI)	11.15525	−16.14029	24	Waterlogged field	88/91	3/91			X			
*COMOROS*												
Mainland-Tanzania												
Dar es Salaam (DAR)	−6.83333	39.26667		Agricultural fields	49/49				X			
Grande Comore												
Boeninidi (BOE)	−11.56592	43.28719	180 m	Indoor drum	6/6				X			
				Outdoor Cistern	53/54			1/54	X			
Bouni (BOU)	−11.48943	43.39748	30 m	Outdoor Cistern Cistern	85/88	1/88		2/88	X			
Malé (MAL)	−11.88647	43.50628	20 m	River bed pools				54/54			X (4)	
				Roadside pools	67/67				X			
Mutsamudu (MUT)	−11.60992	43.39032	20 m	Pools on concrete	80/110	4/110		26/110	X		X (10)	
Ossivo (OSS)	−11.58842	43.27763	170 m	Outdoor cistern	23/32			9/32	X		X (>20)	
Salaman (SAL)	−11.6803	43.2661	20 m	Outdoor Cistern	8/8							
Anjouan												
Assimpao (ASS)	−12.23727	44.31655	0 m	Brackish polluted river	0/2	1/2		1/2	X		X (14)	
				Roadside pools	6/92	6/92		80/92	X		X (10)	
Bambao (BAO)	−12.20143	44.51513	19 m	Water logged field	1/88	6/88		81/88			X (>20)	X (1)
Hohoja (HAJ)	−12.1175	44.48833	0 m	Mountain River bed pools		1/4		3/4			X (1)	
Moya (MOY)	−12.30927	44.43951	0 m	Mountain River bed pools				50/50			X (>20)	
				Swamp	137/138			1/138	X			
Sadapoini (SAD)	−12.37711	44.5012	5 m	Mountain muddy stream		4/96		92/96			X (7)	
Moheli												
Fomboni (FOM)	−12.27690	43.73148	81 m	Roadside pools	104/126			22/126	X			
Hoani (HOA)	−12.25742	43.67292	4 m	Roadside pools	85/124	8/124		31/124	X		X (4)	
Miringoni (MIR)	−12.30198	43.63717	7 m	Sunlit river pools	59/63			4/63	X	X (3)	X (6)	
Ndremeani (NDR)	−12.35487	43.75080	0 m	Roadside pools	101/106			5/106	X			
Wala (WAL)	−12.33825	43.66882	5 m	Brackish lagoon	91/94			3/94	X			
Wanani (WAN)	−12.34511	43.80007	151 m	Muddy pools in field	92/95			3/95	X			

AG *s.l*. Anopheles gambiae s.l. species complex AG *s.s*., *Anopheles gambiae s.s*. -; AM, *Anopheles melas* (Guinea-Bissau)/*Anopheles merus* (Comoros); AA, *Anopheles arabiensis*; AC, *Anopheles coustani*; AP, *Anopheles pretoriensis*; UNK, unknown.

+Numbers in brackets represent number of samples identified.

### Mosquito community sampling

Limited information is available about the mosquito communities on some of the islands, particularly the Comoros. Therefore, we opportunistically collected (adult) and locally reared (larval) mosquitoes at all sites visited for Anopheline sampling and identified specimens using morphological keys when possible. Rearing of larvae to adult stages was also used to aid identification of the Anopheline species present. Representative preserved specimens were deposited in the Bohart Museum at the University of California-Davis. Due to the limited spatial and temporal scale of sampling, these collections should be viewed as representing only a subset of the mosquito community present.

### DNA extraction and species identification

Qiagen blood and tissue kits (Qiagen, Valencia, CA, USA) were used to extract DNA from Anopheline samples using the Qiagen Biosprint 96 system. To distinguish between the different members of the morphologically indistinguishable *An. gambiae s.l*. species complex we used the Scott et al. (1993) PCR assay. We also used a PCR based assay to test for the presence of *Anopheles funestus* complex species amongst larval samples that were not identified as *An. gambiae s.l*. from the Comoros Islands (Koekemoer et al. 2002; Cohuet et al. 2003). The sex of larval samples identified as *An. gambiae s.s*. was determined by PCR (Ng'habi et al. 2007). The molecular form of samples was initially determined using a combination of standard diagnostic assays (Favia et al. 2001; Fanello et al. 2003). However, inconsistencies were found between diagnostics for samples from Guinea-Bissau. As such, we also conducted sequencing or SNP typing of diagnostic sites on the X chromosome, to verify the molecular form, as described in Marsden et al. (2011).

### *Anopheles gambiae* SNP genotyping

SNP discovery was conducted by assessing published sequences representing both the M and S forms from multiple locations in Western (M & S forms), Eastern (S forms) and Southern Africa (S forms) (Morlais et al. 2004; Turner et al. 2005; Slotman et al. 2007; Turner and Hahn 2007; White et al. 2007, 2009, 2010; Cohuet et al. 2008; Mendes et al. 2008; Parmakelis et al. 2008; Santolamazza et al. 2008; Lehmann et al. 2009; Obbard et al. 2009; Harris et al. 2010). The sites used in this article, however, were not included in the SNP discovery stage. Based on these sequences, 96 SNPs were identified, as described in Marsden et al. (2011).

SNP genotyping data for the Guinea-Bissau samples was taken from Marsden et al. (2011), which assayed this genome wide set of 96 SNPs in 323 females using a customized Illumina® Golden Gate assay on the Illumina Bead Station 500G Golden Gate genotyping platform (Illumina, San Diego, CA, USA). However, after excluding loci with high failure rates, poor clustering and those out of Hardy–Weinberg equilibrium, the final data set assessed by Marsden et al. (2011) and used here, consisted of 52 loci which were located on all three chromosomes, with 8–14 SNPs per chromosomal arm.

For the Comoros Islands, we screened 73 of the original set of 96 SNPs assayed by Marsden et al. (2011) and two additional loci (Ag2L-2422654, Ag2L-1272330); the remaining 23/96 SNPs from the Golden Gate assay were excluded as they were uninformative, exhibited poor clusteringand high failure rates for the Guinea-Bissau data set. We selected the Sequenom iPLEX MassARRAY® Sequenom, San Diego, CA, USA platform for genotyping of the Comoros data set as this system requires less input DNA (10 ng/μL) than the Illumina Golden Gate assay (50 ng/μL) and has more flexibility in terms of the number of loci assessed and enables the addition or removal of loci at later time points during the study. For a further comparison of the two different assay platforms see Lee et al. (2012). We designed the multiplex SNP genotyping assay using the Assay Designer module of the MassARRAY Typer 4.0 software package (Sequenom, San Diego, CA, USA), and conducted PCR reactions using Sequenom iPLEX Gold reagent kits following standard procedures at the Veterinary Genetics Laboratory, University of California-Davis. To verify consistency in SNP calling between the Illumina and Sequenom genotyping platforms, we screened 14 samples on both systems and found 97% of genotype calls (839/866) to be consistent (excluding 11 loci that failed or could not be clustered, based on an assessment of the complete data set).

### Analyses of SNP data

Within each study area, we calculated genetic diversity metrics separately for each site, and also for each molecular form. Specifically, we calculated expected heterozygosity (*H*_e_) using GenALEX6.3 (Peakall and Smouse 2006), and allelic richness standardized for sample size (*R*_S_) as estimated by FSTAT 2.9.3.2 (Goudet 1995).

We evaluated population structure and gene flow amongst sites using two approaches. We first calculated *F*_ST_ using Arlequin 3.5.1.2 (Excoffier and Lischer 2010), with significance adjusted for an alpha value of 0.05 according to Bonferroni correction. *F*_ST_ is a measure of genetic differentiation based on allele frequency differences between populations with values theoretically ranging from 0 (no differences in allele frequencies, panmictic populations) to 1 (populations share no alleles, no gene flow; Hartl and Clark 2006). A limitation of *F*_ST_ is that it assumes populations have reached equilibrium between mutation and migration, which is not applicable for recently bottlenecked, founded or isolated populations (Whitlock and McCauley 1999). In cases where these assumptions are violated, *F*_ST_ estimates may be biased, and other types of analyses may be more appropriate (Pearse and Crandall 2004). Therefore, we evaluated population structure and gene flow using the individual-based Bayesian clustering algorithm implemented in the programme STRUCTURE v2.3.3 (Prichard et al. 2000) which calculates the number of genetic populations (clusters) within a data set as well as the ancestry for each individual to the different clusters thus enabling detection of population structure and recent migrants, without assumptions about migration rates, population sizes or mutation-drift equilibrium. However, we include results from both *F*_ST_ and STRUCTURE analyses, as it has been shown that consistent results from different analyses of the same data set, can give more certainty that findings reflect a real signal rather than a spurious one resulting from invalid assumptions associated with specific analysis approaches (Pearse et al. 2006).

### ND5 and internal transcribed spacer (ITS) sequencing

Where populations were found to be isolated based on SNP data, we further evaluated isolation and investigated the origin of island populations, by sequencing the nuclear ITS and mitochondrial NADH dehydrogenase subunit (ND5) gene, which have been evaluated in *An. gambiae* populations across Africa (reviewed in Marshall et al. 2008). The ITS region was amplified with primers 28S_Rev and 18S_For (Gentile et al. 2001), and ND5 amplified with primers 19CL and DMP3A (Besansky et al. 1997). The presence of amplified DNA was confirmed using a QIAxcel electrophoresis system (Qiagen) and PCR reactions were cleaned up with ExoSAP-it (Affymetrix, Santa Clara, CA, USA) and sequenced on an ABI3070 at the DNA Sequencing Facility at UC Davis with the abovementioned primers.

Sequences were edited and aligned to create haplotypes using the programme Geneious 5.3.6 (Biomatters, Auckland, New Zealand). These haplotypes were then cross-referenced with published sequences from East African populations in order to detect novel haplotypes and to resolve the genetic relationship between the islands and mainland Africa (ITS – Della Torre et al. 2001; Gentile et al. 2001, 2002; Marshall et al. 2008 and ND5 Besansky et al. 1997; Lehmann et al. 1997; Donnelly et al. 2001, 2004). The programme Network 4.610 (Fluxus Technology, Kiel, Germany) was used to construct haplotype networks for the ND5 gene. ND5 haplotype (*h*) and sequence diversity (*π*) were calculated using DnaSP v5 (Librado and Rozas 2009).

## Results

### Mosquito community sampling

In Guinea-Bissau, 20 mosquito species from the *Aedes*, *Anopheles, Coquillettidia, Culex, Mansonia* genera were found (11 on the islands, 13 on the mainland; Tables S1–S3). All species have previously been recorded in the region, and no unique morphological characteristics were found to be associated with island specimens. However, it is noteworthy that our collections were made from a limited number of habitats and at a single time point.

In the Comoros 21 mosquito species representing five genera (*Aedes*, *Eretmapodites*, *Culex*, *Anopheles*, *Lutzia*) were collected in the habitats from which we sampled. Full taxonomic records are detailed in Tables S4–S7. However, four records were of particular note. On the island of Anjouan, we collected a single specimen of an Anopheline (Table S6) resembling no described mosquito species described from mainland Africa (Gillies and De Meillon 1968; Gillies and Coetzee 1987) and Madagascar (Doucet 1951). Our collections also found that the wing markings of *Anopheles pretoriensis* Theobald samples consistently varied from those on continental Africa (Table S6; Fig. S1), which may reflect local divergence. We also recorded a specimen which we named *Cx. sunyaniensis* like, that shared characters of *Cx. (Eum.) sunyaniensis* Edwards and *Cx. (Eum.) wigglesworthi* Edwards, neither of which have been recorded in the Comoros Islands (Table S5). Lastly, we collected several male and female *Aedes (Steg.) albopictus* Skuse*,* providing the first record of this important arbovirus vector on the island of Anjouan (Calisher et al. 1981; Delatte et al. 2008; *Ae. albopictus* was recorded on the nearby island of Mayotte in 2001, Girod 2004).

### Anopheles species composition

#### Guinea Bissau

In total, we assayed the species composition of 440 Anopheline samples from Guinea Bissau. We detected two major Anopheline malaria vectors; *An. gambiae s.s*. which was the dominant species (95%, *n* = 418), and the salt water tolerant species, *Anopheles melas*, which was relatively rare (5%, *n* = 22). The 418 *An. gambiae s.s*. samples consisted of both the M (*n* = 57) and S (*n* = 244) molecular forms, as well as hybrids (*n* = 117, Marsden et al. 2011). Only a single specimen of *Anopheles arabiensis* was detected amongst the 439 samples (verified in three independent PCR's, Table 1).

#### Comoros

We assayed 1497 Anopheline like larvae from the Comoros Islands using species diagnostic PCR to detect members of the *An. gambiae s.l*. (Scott et al. 1993) and *An. funestus* species complex (Cohuet et al. 2003). On each of the islands, we detected two major malaria vectors; *An. gambiae s.s*. (67%, *n* = 998) which was common, and the saltwater tolerant, *Anopheles merus*, which was rare (2%, *n* = 31; Table 1). Both species have been previously recorded in the Comoros, although it is noteworthy that *An. merus* had only been described on Moheli (and Mayotte) (Julvez and Mouchet 1996). Consistent with other studies (Julvez and Mouchet 1996), *An. gambiae* larvae on the Grande Comore were found in non-classical breeding sites, such as outdoor water cisterns and indoor water containers, due to the highly permeable volcanic soil which results in a lack of surface water (Fig. S2; Table 1). All *An. gambiae s.s*. samples were shown to represent the S molecular form, based on the Favia diagnostic (Favia et al. 2001).

Contrary to published records as recent as 2003 (Ayala et al. 2006), we did not detect the presence of *An. funestus* with either PCR assays or morphological assessments. This likely reflects the fact that we were collecting larvae, and that *An. funestus* larval sites are ‘notoriously difficult’ to find (Gillies and De Meillon 1968) and were not specifically targeted in this survey. A considerable number of Anopheline like larvae (*n* = 468) were not identified by PCR to be members of either the *An. gambiae s.l*. complex or *An. funestus* species group, particularly on Anjouan (308/470, Table 1). However, many reared larvae were morphologically identified to be *An. pretoriensis* (Theobald), which has previously been recorded on all of the islands (Julvez and Mouchet 1996), but is almost entirely zoophillic and not considered a vector of malaria (Gillies and De Meillon 1968; Julvez and Mouchet 1996). Overall, morphological identification of adults reared from larvae showed the presence of *An. pretoriensis* at 5/5 sites on Anjouan, 3/6 sites on Moheli and 3/6 sites on Grande Comore (Table 1). All other reared larvae were identified as *An. gambiae s.l*., with the exception of three *Anopheles coustani* (Lavaren) found at Miringoni on Moheli which also has previously been recorded on the Comoros (Julvez and Mouchet 1996).

### Genetic diversity and differentiation

#### Guinea-Bissau

**Figure 1:**
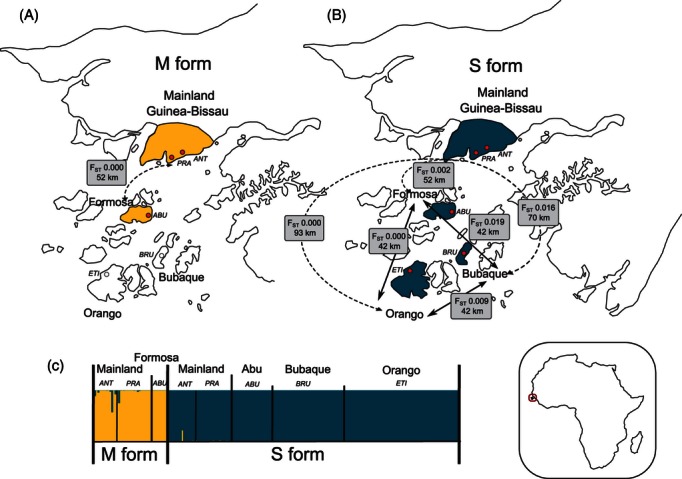
Map of Guinea Bissau study area drawn to scale, with pairwise geographical and genetic distances (*F*_ST_) between the mainland and islands shown in grey boxes for M form (A) and S form (B) populations. Sampling sites are depicted with red circles with abbreviations taken from Table 1. The mainland and island colours correspond to cluster membership resulting from the STRUCTURE analysis depicted in C. (C) Clustering analyses using STRUCTURE detected two clusters within the data set corresponding to the M and S form, represented here by two colours. Columns represent individuals with colours depicting the proportion of their genome assigned to the different genetic clusters.

Prior to genetic analyses of the Guinea-Bissau data set, we removed samples with >10% missing data and sites represented by fewer than eight samples. Furthermore, we excluded all samples designated as M-S form hybrids (*n* = 103) as previous assessments showed the hybrids to consist of a range of backcrosses, and thus could not be classed as a ‘population’ that could be assessed separately. The final data set consisted of 52 loci for 213 samples including 141 samples from the Bijagós archipelago (Formosa, *n* = 31; Bubaque, *n* = 43; Orango, *n* = 68), and 72 samples from the coastal region of mainland Guinea-Bissau (Antula, *n* = 30; Prabis *n* = 42). Genetic diversity metrics for the M form (*R*_S_ and *H*_E_) were found to be slightly higher on the island of Formosa than on the mainland, whereas they were similar between mainland and island populations of the S form (Table 2). Despite being separated by distances of 42–103 km, genetic differentiation between the island and the mainland sites in Guinea-Bissau was low for both the M and S forms of *An. gambiae* (F_ST_ 0-0.016; *P* > 0.05; Fig. 1A,B). M form samples were absent from Orango island and very rare on Bubaque island (*n* = 2), so we could not assess *F*_ST_ among M form island populations. S form samples were collected from all three islands, and genetic differentiation was found to be low among these sites (F_ST_ 0-0.019; *P* < 0.05; Fig. 1B). Consistent with the *F*_ST_ results, clustering analyses conducted in STRUCTURE and assessed with the Δ*K* statistic (Evanno et al. 2005), showed the most likely number of clusters within the Guinea-Bissau data set to be two, corresponding to the M and S forms (Fig. 1). We subsequently ran STRUCTURE on the M and S samples independently to assess for additional genetic sub-division (data not shown). However, we found no evidence of further structure, suggesting that for each form, the island and mainland samples are derived from represent a single genetic population.

**Table 2:** Genetic diversity statistics for mainland and island sites

Site	*R_S_	*H*_E_
*Guinea-Bissau (M form)*		
**Mainland**		
Antula-Prabis (*n* = 35)	1.469 (0.054)	0.148 (0.024)
**Island**		
Formosa (*n* = 8)	1.317 (0.064)	0.110 (0.025)
*Guinea-Bissau (S form)*		
**Mainland**		
Antula-Prabis (*n* = 59)	1.560 (0.057)	0.182 (0.025)
**Islands**		
Formosa (*n* = 23)	1.541 (0.057)	0.176 (0.025)
Bubaque (*n* = 43)	1.558 (0.055)	0.193 (0.027)
Orango (*n* = 67)	1.562 (0.053)	0.186 (0.025)
*Comoros (S form)*		
**Mainland-Tanzania**		
Dar-es-Salaam (*n* = 49)	1.868 (0.029)	0.187 (0.029)
**Islands**		
Grande Comore (*n* = 109)	1.680 (0.036)	0.207 (0.036)
Moheli (*n* = 149)	1.624 (0.036)	0.201 (0.036)
Anjouan (*n* = 66)	1.600 (0.034)	0.190 (0.034)

* RS = allelic richness standardised for sample size, H_E_ = expected heterozygosity

#### Comoros

We assayed 75 SNPs in female *An. gambiae s.s*. samples from the Comoros Islands and Tanzania. After excluding loci that were monomorphic, out of Hardy–Weinberg equilibrium in more than one population and/or exhibited high failure rates or poor clustering, 31 loci remained for analysis. The 31 loci were located across all chromosomal arms; 7 on chromosome 2L, 7 on 2R, 9 on 3L, 5 on 3R, 3 on X (Table S8). We excluded samples with more than 10% missing data and samples from sites with less than eight samples, leaving 373 samples from the islands of Grande Comore (*n* = 109), Anjouan (*n* = 66) and Moheli (*n* = 149) and mainland Tanzania (*n* = 49). It is noteworthy that all samples from the site of Malé on Grande Comore had to be excluded due to DNA degradation.

**Figure 2:**
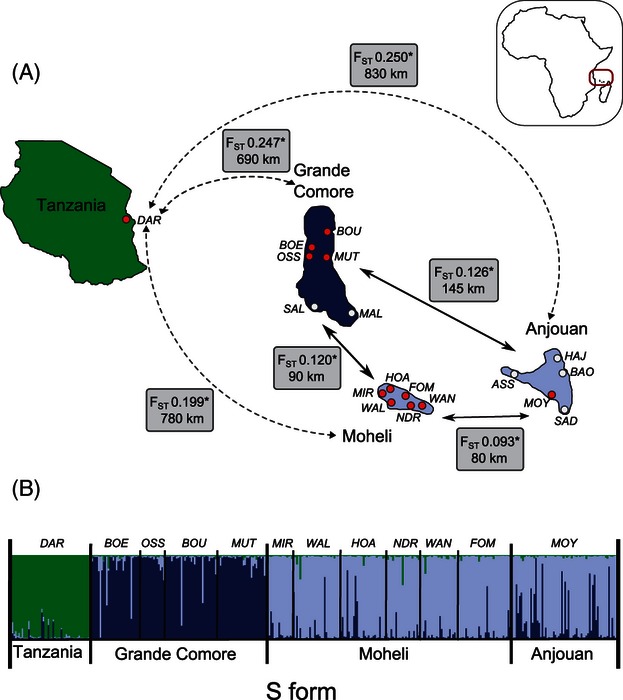
(A) Map of study area in Tanzania and the Comoros Islands. Tanzania is not drawn to scale. Sampling sites are depicted with circles with abbreviations taken from Table 1. Sites used in genetic analyses are shown in red. Grey boxes show pairwise geographical and genetic distances (*F*_ST_) between the mainland and islands. The mainland and island colours correspond to cluster membership resulting from the STRUCTURE depicted in B. (B) Clustering analyses using STRUCTURE detected three clusters within the data set, represented here by three colours. Columns represent individuals with colours depicting the proportion of their genome assigned to the different genetic clusters.

Due to differences in the specific SNP markers that were screened and/or successfully assayed, a direct comparison of diversity levels between the Comoros and Guinea-Bissau was not possible. However, within the Comoros data set we found allelic richness to be consistently lower on the island than mainland sites, whereas *H*_E_ estimates were similar. Pairwise comparisons detected significant genetic differentiation between each of the Comoros Islands and mainland Tanzania (*F*_ST_ 0.199–0.250, *P* < 0.05; Fig 2A), with the greatest differentiation found between the mainland and the most distant island, Anjouan (*F*_ST_ 0.250, 145 km; Fig. 2A). Examination of locus-specific *F*_ST_ values showed this differentiation was not an artefact of a small number of loci with large *F*_ST_ values. However, *F*_ST_ values were highly variable and not all loci were significantly differentiated (10–16 per island; Table S8). This is not unusual for *An. gambiae* (Turner et al. 2005; Marsden et al. 2011), and not unexpected if the islands were recently isolated from the mainland.

Genetic differentiation was also high and significant amongst the three islands (*F*_ST_ 0.093–0.126, Fig. 2A), although it was lowest between the two closest islands, Moheli and Anjouan (*F*_ST_ 0.120, 80 km), and highest between Anjouan and Grande Comore which are separated by the greatest distance (*F*_ST_ 0.126, 145 km). No differentiation was observed amongst the six sites within Moheli island (*F*_ST_ 0–0.013, *P* > 0.05), whereas significant *F*_ST_ was found between the site of Bouni (BOU) and the three other sites on Grande Comore (0.044–0.071, *P* < 0.05; Fig. S3). With *An. gambiae* s.s. detected at only a single site, differentiation amongst populations could not be assessed on Anjouan.

Amongst the Tanzanian and Comoros samples, STRUCTURE analyses detected three clusters corresponding to: (i) Mainland Tanzania; (ii) Grande Comore; (iii) Moheli and Anjouan (Fig. 2B). We found no evidence of population structure between the islands of Anjouan and Moheli, or between sites within islands. We did not detect any recent migrants between the mainland and island sites using STRUCTURE, as would have been indicated by high assignments (>70%) of mainland samples to an island cluster, or vice versa (Fig. 2B). Together these data suggest that there are barriers to gene flow between the Comoros Islands and mainland Tanzania. In contrast, our analyses detected a number of migrants between the clusters Anjouan–Moheli and Grande Comore, as illustrated by a sample that was assigned to a different cluster to the one it had been sampled from (e.g. 3/109, 2.8%, Grande Comore samples assigned to the Moheli-Anjouan cluster and 9/215, 4.2%, Moheli-Anjouan samples assigned to the Grande Comore cluster, using a cutoff value of 70%). Such a pattern is suggestive of some ongoing gene flow amongst the islands.

### Internal transcribed spacer and ND5 haplotype data

We further investigated isolation of the Comoros islands *An. gambiae* population by sequencing the ITS and ND5 genes. Previous studies detected limited variation at the ITS (Marshall et al. 2008). Therefore, we screened a subset of 30 samples; 5 from Tanzania and ten samples from Grande Comore, 10 samples from Moheli and five from Anjouan. All sequences were found to represent the S form haplotype IA, which is one of the two common S form ITS haplotypes (Marshall et al. 2008). Due to the high frequency and widespread distribution of the IA haplotype across Eastern Africa (Madagascar, Kenya, Malawi and Tanzania; Le Goff et al. 2006; Marshall et al. 2008) these data were not informative about the origin of the Comoros population. Nonetheless, it is noteworthy that the most common S haplotype, 1D, and the only haplotype recorded to date in Mozambique (Marshall et al. 2008), was absent amongst in the Comoros islands.

Due to the higher levels of variation in the ND5 gene in comparison to the ITS (e.g. Marshall et al. 2008), we screened a larger number of samples at this region (*n* = 183): 31 from Bouni and 31 from Bouenindi (Grand Comore); 29 from Wala and 30 from Wanani (Moheli); 30 from Moya (Anjouan); and 32 from Dar es Salaam (Tanzania). The ND5 sequences generated in this study were ∼150 bp longer (811 bp) than those deposited in Genbank (665 bp), which yielded three extra variable sites. To utilize this additional information as well as published sequence data, we conducted analyses on two ND5 data sets; the first using the full-length sequences (811 bp) generated for the samples screened in this study; and the second using the sequences from this study trimmed to 665 bp and combined with published sequences from East Africa.

**Figure 3:**
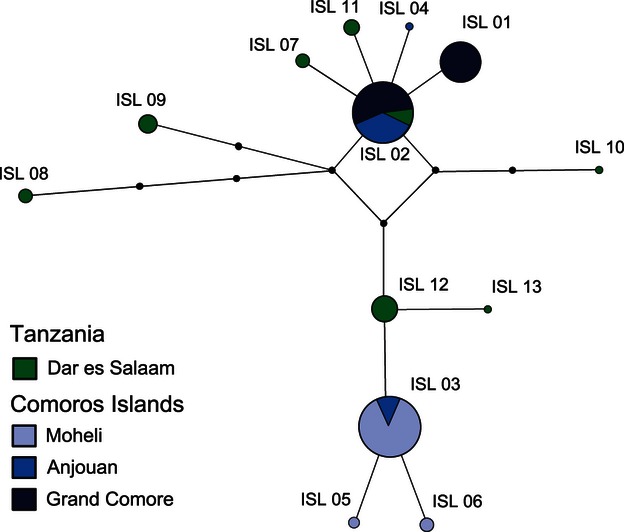
Mitochondrial ND5 haplotype network based on full-length (811 bp) sequences detected in this study. Circles represent haplotypes and are coloured according to sampling location, and sized proportional to haplotype frequency. Black nodes denote unsampled haplotypes.

We detected 13 full-length ND5 sequences; eight in Dar es Salaam Tanzania, and six from the Comoros islands (Fig. 3, Table S9). Only a single ND5 haplotype (ISL 02) was shared between Tanzania and the Comoros islands, which is indicative of severely restricted gene flow (Fig. 3). Furthermore, the islands showed lower ND5 diversity (*n* = 30–62, two to three haplotypes, π < 0.00175, *h* < 0.0018) relative to the mainland (Dar es Salaam, Tanzania, *n* = 32, eight haplotypes, π = 0.00395, *h* = 0.0040) which may be explained by stronger genetic drift in isolated populations due to bottlenecks and/or smaller Ne. The lower ND5 diversity may also reflect that there were few colonization events from the mainland. We detected two to three of the six haplotypes on each island. No haplotypes were shared across all three islands, but the two most common haplotypes were each detected on more than one island; ISL02 was detected on Grande Comore and Anjouan, and ISL03 between Anjouan and Moheli (Fig. 3). These data suggest that there is ongoing but restricted gene flow amongst the islands.

Trimming of the ND5 sequences condensed the six Comoros haplotypes into four, but did not affect the number of haplotypes in Dar es Salaam (8; Table S10). Two of the four Comoros haplotypes, H32 which was found on Grande Comore (*n* = 62/63) and Moheli (*n* = 23/30), and H11 which was found on Anjouan (*n* = 56/59) and Moheli (*n* = 6), have previously been detected in Malawi, Kenya and Tanzania (Fig S5, Besansky et al. 1997; Lehmann et al. 1997; Marshall et al. 2008). Due to the widespread distribution these haplotypes, these data are not informative about the mainland origin of the Comoros islands. The remaining two Comoros haplotypes were rare and unique to this locality (ISL04 on Moheli, *n* = 1; ISL06 on Anjouan, *n* = 3). These private haplotypes were similar to the two common island haplotypes (ISL04 differed by one base pair from H11 and ISL06 by a single base pair H32) indicating that they may represent mutation events that occurred on the Comoros islands post colonization, which is consistent with isolation of the islands from mainland Africa. However, more widespread sampling of Africa is required to verify this.

## Discussion

Considerable advances have been made in recent years with regards to the development of transgenic Anopheline mosquitoes refractory to the malaria parasite (Alphey 2009). However, before such an approach can be widely implemented, it is critical to evaluate risks associated with GMM through rigorous scientific investigation and to demonstrate proof of principle of such technologies in this system. Oceanic islands, in particular, have been highlighted as potentially suitable sites for such trials, as they should be isolated from mainland sites due to the natural barrier created by water. Here, we genetically evaluated the suitability of the Bijagós archipelago and the Comoros Islands, for GMM releases.

### Bijagós archipelago

We assessed three islands of the Bijagós archipelago located off the coast of Guinea Bissau in West Africa, where there is active and ongoing malaria transmission (WHO 2011). *Anopheles gambiae s.s*. was found to be present alongside *An. melas,* at all sites in Guinea-Bissau (Petrarca et al. 1983; Jaenson et al. 1994). On the islands of Bubaque and Orango, the relative abundance of these two vectors is difficult to assess, as collections were largely comprised of larvae which were specifically collected from typical *An. gambiae s.s*. larval habitat sites. However, *An. gambiae* was the dominant species on the mainland and the island of Formosa where only indoor resting adult mosquitoes were collected (Table 1). For a GMM release site, this is advantageous, as it is preferable for the target species to be the dominant malaria vector (Malcolm et al. 2009).

The Bijagós islands we assessed are separated from mainland Guinea-Bissau by 52–93 km. However, despite being separated by distances exceeding the natural dispersal capabilities of *An*. *gambiae s.s*. (7 km), we found no evidence of genetic sub-division between the island and mainland sites in either the M or S forms, suggesting there is considerable gene flow (Fig. 1). Consistent with this, in the S form we found no evidence of a reduction in genetic diversity which might be expected for a genetically isolated island population. In the M form, diversity was lower on Formosa; however, this site was represented by <10 samples, and thus is likely a sampling effect. We suggest that the genetic connectivity we observed in the Bijagós may be explained by ‘island hopping’ between the many islands of the archipelago, most of which are located <10 km apart, and some of which are located <10 km from the mainland (Fig. 1). Specifically, we hypothesize that rather than direct dispersal between the mainland and far islands (∼50–90 km), there is dispersal between the mainland and closest island, and then the closest island and nearby islands, which are separated by much smaller distances (<10 km). Alternatively, or additionally, there may be inadvertent movement of mosquitoes via the high volumes of human local boat traffic between the mainland and island sites as well as amongst the islands themselves, which is important for trade, transportation and fishing. These findings are consistent with the low UNEP isolation index for the Bijagós islands we assessed (10 – Formosa, Bubaque; 17 – Orango), which reflects not only the distance of an island to the continent but also to the other islands. Overall, we suggest the lack of isolation between the Bijagós archipelago and mainland Guinea-Bissau makes them an unsuitable site for a GMM release. In addition, the presence of both the M and S forms as well as the atypical high rates of hybridization between them, present unique challenges for implementation of an isolated GMM trial at this site. Specifically, previous genetic studies have shown that hybridization results in highly asymmetric introgression from the M form into the S form (Marsden et al. 2011). It is uncertain how modified genes would move between the M and S forms under these patterns of gene flow. However, it is possible that if only the S form was modified, genetically modified genes would be unlikely to spread into the M form, whereas if the M form was modified, genes would spread into the S form (Marsden et al. 2011).

### Comoros Islands

A number of vector borne diseases are transmitted on the Comoros Islands including dengue fever, (Gautret et al. 2010), chikungunya virus (Sergon et al. 2007), rift valley fever (Sissoko et al. 2009) and malaria (Rebaudet et al. 2010). We found vectors for these diseases on each of the islands (Table S1–S4), including the first record of *Ae. albopictus* on Anjouan (vector of Chikungunya and Dengue fever virus). However, ongoing malaria transmission presents the highest health burden on the islands (WHO 2011). We detected two primary malaria vectors, *An. gambiae s.s*. and *An. merus*. Unfortunately, the relative abundance of these vectors is difficult to assess due to the bias in sampling efforts towards atypical peridomestic, but known *An. gambiae s.s.,* larval habitat sites (Julvez and Mouchet 1996).

In comparison to the Bijagós archipelago, the Comoros Islands are considerably more physically isolated (∼700–800 km vs <100 km) resulting in a higher isolation index (49, UNEP 2010). We found *An. gambiae s.s*. from the three islands (Grande Comore, Moheli, Anjouan) to be derived from different genetic populations of mosquitoes from mainland Tanzania, and found no evidence of recent migrants between the mainland and islands amongst our samples (Fig. 2), which is consistent with isolation of the islands.

The presence of multiple private ND5 haplotypes on the Comoros islands (5/6 full-length sequences, 2/4 trimmed sequences) was also suggestive of isolation between the islands and mainland. Furthermore, as expected for isolated populations, genetic diversity estimates based on SNP data (Table 2) and ND5 sequences (Table 3) were lower on the islands than the mainland, with the exception of SNP based *H*_E_ estimates which were similar on the mainland and islands despite lower allelic richness on the islands, which in itself is suggestive of a recent bottleneck (Table 2). Lastly, the unique morphological features of some taxa recorded during the mosquito community sampling on the Comoros Islands is also consistent with isolation of these islands (e.g. unknown Anopheline species, wing pattern divergence of *An. pretoriensis,* Tables S4–S7). Together, these data suggest that the Indian Ocean presents a significant barrier to gene flow between the mainland and Comoros islands and that human-assisted movement of mosquitoes is likely to be minimal. As such, in terms of isolation, the Comoros Islands appear to be good potential candidate sites for GMM release trials as our data suggests there is a low risk of GMM escapees reaching mainland Africa.

**Table 3:** Tanzania and Comoros ND5 diversity based on full-length sequences (811 bp)

Population	Number of haplotypes	Haplotype diversity (*h*)	Sequence diversity (*π*)
*Mainland-Tanzania*			
Dar-es-Salaam (*n* = 32)	8	0.843	0.0040
*Comoros Islands*			
Grand Comore (*n* = 62)	2	0.500	0.0006
Anjouan (*n* = 30)	3	0.384	0.0017
Moheli (*n* = 59)	3	0.161	0.0002

Despite this evidence of restricted movement, *F*_ST_ values were not indicative of complete genetic isolation between the Comoros islands and mainland (*F*_ST_ = 1). This could be the result of *F*_ST_ not reflecting the true level of isolation. For example, if the Comoros islands were colonized relatively recently, there may have been insufficient time for differentiation to occur despite the absence of any ongoing gene flow (non-equilibrium population). This is consistent with ND5 haplotype data which shows the presence of both unique and shared haplotypes. Or, levels of divergence may have been underestimated due to the absence of Comoros samples in the SNP ascertainment panel, which would have resulted in under-representation of polymorphisms unique to the Comoros. Another explanation is that an *F*_ST_ value of <1 may reflect that there is ongoing gene flow between the mainland and islands sites. Indeed, population bottlenecks can cause rapid divergence in allele frequencies resulting in elevated *F*_ST_ values despite high gene flow as the population is not at equilibrium. In these situations, methods that do not assume mutation-equilibrium, such as STRUCTURE, are more appropriate. As stated above, STRUCTURE analyses and the distribution of ND5 haplotypes showed strong genetic structuring between mainland and island sites, indicating gene flow is restricted. Moreover, amongst our samples we found no evidence of recent migrants. As such, our data do not appear to be consistent with a hypothesis of elevated *F*_ST_ despite high gene flow due to a bottleneck. However, there may well be a low level ongoing migration which our sampling regime failed to detect. Additional sampling from the Comoros, sites along coastal East Africa (e.g. Northern Mozambique, ∼300–450 km from the islands) and Madagascar (400–450 km), as well as sampling from transport locations (airports and ports) would be useful to quantify any migration, particularly *from* the Comoros islands.

Given that *An. gambiae* is always found alongside human populations which may inadvertently transport individuals, there will always be some risk of migration and thus complete isolation of field site is unrealistic. General guidelines concerning acceptable levels of risk have not been developed as these will be specific to the species, trial conditions (e.g. fitness of the GMM) and technology being applied: A low level of migration would pose a smaller risk for self-limiting approaches (versus self-propagating see Introduction), such as are available for *An. gambiae*. For this reason, mathematical models would be useful in assessing the potential risk posed by migration on a case by case basis (e.g. Marshall and Hay 2012). Moreover, risk could be reduced by implementing measures to prevent human-assisted movement of vectors (insecticide spraying of aeroplanes) and a monitoring programme to detect escapees (e.g. fluorescent markers, James 2005).

If a GMM trial were to be conducted in the Comoros, a single island would need to be selected. The question of which of the islands is the most suitable for a GMM release requires consideration of genetic isolation amongst and within the islands. Flights, ferries and local boats result in the movement of people, and thus potentially mosquitoes, between the three islands, which are separated from each other by between 80 and 145 km. We found the greatest genetic differentiation between Grande Comore and Anjouan (*F*_ST_ = 0.126), indicating that human-assisted movement is lowest between the islands which are located the furthest apart (145 km; Fig. 2). Interestingly, this is consistent with population structure analyses of *Plasmodium falciparum* in the Comoros, which found differentiation of parasite populations to be highest between this pair of islands (Rebaudet et al. 2010). We found differentiation to be lowest between *An. gambiae* s.s. from Moheli and Anjouan (*F*_ST_ = 0.093), which were shown to constitute a single genetic population (Fig. 2). Overall, Grande Comore was the only island found to be genetically distinct from the other islands, although it should be noted that there was evidence of some ongoing gene flow between Grand Comore and Moheli and Anjouan (Fig. 2). In terms of a GMM release, evidence that gene flow between Grande Comore and the other islands is restricted is advantageous, as it reduces the risk of spread of escapees to other islands, as well as the immigration of mosquitoes onto the island.

Population structure within a release site, that is, an island, has the potential to inhibit GMM programmes as genetic sub-division may impede the spread of genes across the target population if a genetic drive system is linked to an effector gene so to facilitate the rapid spread of modified genes through the population. However, in taxa such as *An. gambiae,* where genetic drive technology is not yet available, or in instances where sterile insect technologies are used, inundative release (release of enormous numbers of modified mosquitoes) will be necessary (Benedict 2011). In these cases, population structure is advantageous as sub-populations can be used to enable a release programme to be conducted in sections (e.g. as recommended for *An. arabiensis* on Reunion island, Malcolm et al. 2009). We found no evidence of sub-structuring within the island of Moheli, and on Anjouan only a single site was assessed. However, on Grande Comore, the most north-eastern site (Bouni), showed evidence of elevated differentiation (Fig. S3). This may be related to topography (Fig. S4), and it is possible these naturally isolating barriers could be enhanced to create an isolated site within Grande Comore. Alternatively, the topography of the most southern section of the island, indicate that this region may be isolated (Fig. S4). Indeed, our surveys detected *An. gambiae* in this region (Malé), but samples were degraded and unsuitable for SNP analysis, and therefore genetic evaluations of mosquitoes from this area would be useful to assess isolation.

In addition to genetic considerations, other island characteristics, such as island size, political stability and logistical considerations, are important. Whilst a full evaluation of these factors is outside of the scope of this study and thus not specifically investigated here, it is noteworthy that the highly mountainous terrain and political instability of Anjouan, make this island less appealing as a release site. In contrast, the smaller size (290 km^2^) and relatively flat terrain of Moheli, make this island an attractive prospect if trying to conduct an island wide implementation.

Within the range of *An. gambiae*, there are only a small number of island sites potentially suitable for field trials*,* and only a few of these have been assessed (e.g. Pinto et al. 2002; Moreno et al. 2007). Nonetheless, our findings are broadly consistent with studies of a volcanic chain of four widely separated islands (≥160 km apart) in the Gulf of Guinea (Bioko, São Tomé, Príncipe and Annobón). Specifically, Bioko Island which is isolated to a similar degree to the Bijagós ∼75 km off the coast of Cameroon and with an UNEP isolation index of 17, was found not to be genetically isolated from the mainland (Moreno et al. 2007). In contrast, the islands of São Tomé, Príncipe and Annobón, which are located 240–350 km from mainland Gabon and with similar UNEP isolation indices (39–45) to the Comoros (49), were found to be genetically isolated from mainland populations (Pinto et al. 2002; Moreno et al. 2007). Together these data suggest that island sites with an isolation index of <20, are likely not worthy of investigation as potential GMM field trial sites for *An. gambiae*.

## Conclusion

We evaluated *An. gambiae* populations from two oceanic island groups for evidence of genetic isolation from mainland populations to evaluate their potential for use in GMM releases. Our data suggest that the three islands from the Bijagós archipelago were not genetically isolated from mainland populations. In contrast, genetic isolation was found between the Comoros Islands and mainland Tanzania, thus highlighting these islands as sites worthy of further investigation for GMM trials. The relative isolation of Grande Comore, as well as the presence of population sub-division within the island, suggests that it may be the most suitable trial site for existing GMM technologies. However, before proceeding further, it would be valuable to: (i) conduct additional sampling from the Comoros, mainland Africa and Madagascar, including some transport locations (airports and ports), to further evaluate the risk of escapees; (ii) evaluate the distribution of populations, and their genetic isolation, in the most southern region of Grande Comore.

## Data archiving

Data for this study has been deposited in the open source online vector database *PopI*: (https://grassi2.ucdavis.edu/) OpenProjects – IslandEcology page, where all other ecological and genetic information associated with these samples is also available. ND5 sequence data were deposited to GENBANK: accession numbers KC249505-517.

## Supporting Information

**Figure S1.** Sketch of the wing markings of *Anopheles pretoriensis* collected from the Comoros Islands.

**Figure S2.** Sampling from atypical peridomestic larval habitats on the island of Grande Comore: Top-cisterns (Bouni), Bottom-indoor water drum (Boeindi).

**Figure S3.** Pairwise *F*_ST_ values for sites within islands (above diagonal) and geographic distances (below the diagonal).

**Figure S4.** Mitochondrial ND5 haplotype network based on trimmed length (665 bp) sequences detected in this study combined with published sequences from East Africa (Besansky et al. 1997; Lehmann et al. 1997; Donnelly et al. 2001, 2004).

**Figure S5.** Elevation map for Grande Comore.

**Table S1.** Location and habitat characteristics of *Anopheles* on the Bijagós archipelago.

**Table S2.** Location and habitat characteristics of *Aedes* and other genera on the Bijagós archipelago.

**Table S3.** Location and habitat characteristics of *Culex* and other genera on the Bijagós archipelago.

**Table S4.** Location and habitat characteristics of *Aedes* mosquitoes collected in Comoros islands.

**Table S5.** Location and habitat characteristics of *Culex* mosquitoes collected in Comoros islands.

**Table S6.** Location and habitat characteristics of *Anopheles* mosquitoes collected in Comoros islands.

**Table S7.** Location and habitat characteristics of *Eretmapodites* and other mosquitoes collected in Comoros islands.

**Table S8.** Locus specific pairwise *F*_ST_ values for Tanzania-Comoros island comparisons, with significant values after Bonferoni correction underlined.

**Table S9.** Frequency of full length ND5 haplotypes by sampling location.

**Table S10.** Haplotype numbers from Fig. S4 and their corresponding haplotype designations by Besansky and Genbank accession numbers.
