# Supplementary material for: An analysis of two island groups as potential sites for trials of transgenic mosquitoes for malaria control
Source: Evol Appl. 2013 Feb 22;6(4):706–20. doi: 10.1111/eva.12056 (PMC3684749; doi:10.1111/eva.12056)
Supplement: Supplementary file 1 [file eva0006-0706-SD1.pdf]

Figure S1: Sketch of the wing markings of *Anopheles pretoriensis* collected from the Comoros Islands. Markings consistently varied from that described by Gillies and DeMeillon (1968). Specifically, in Comoros specimens the 4th pale area (from wing base) of the 1st vein is in line with the 4th pale spot of costa so that the 2nd main dark area has only one pale interruption. In mainland African specimens the 2nd main dark area of 1st vein has two pale interruptions. Additionally, the 2nd pale area of vein 3 is often absent in Comoros specimens.

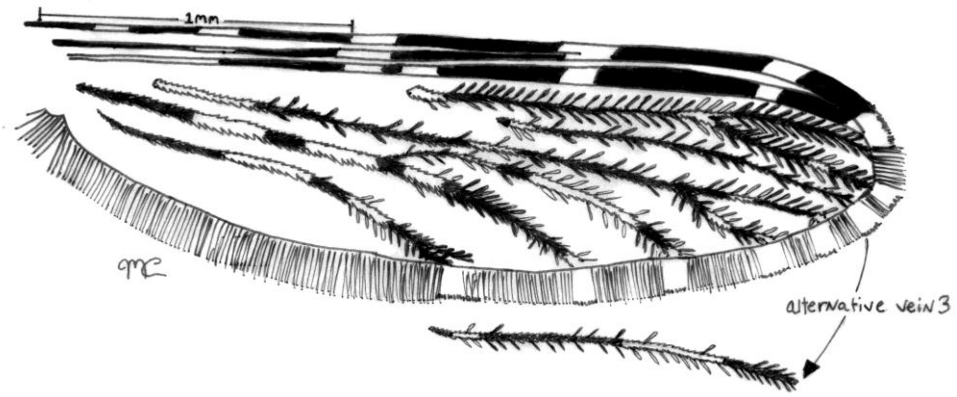

Figure S2: Sampling from atypical peridomestic larval habitats on the island of Grande Comore: Top-cisterns (Bouni), Bottom-indoor water drum (Boeindi).

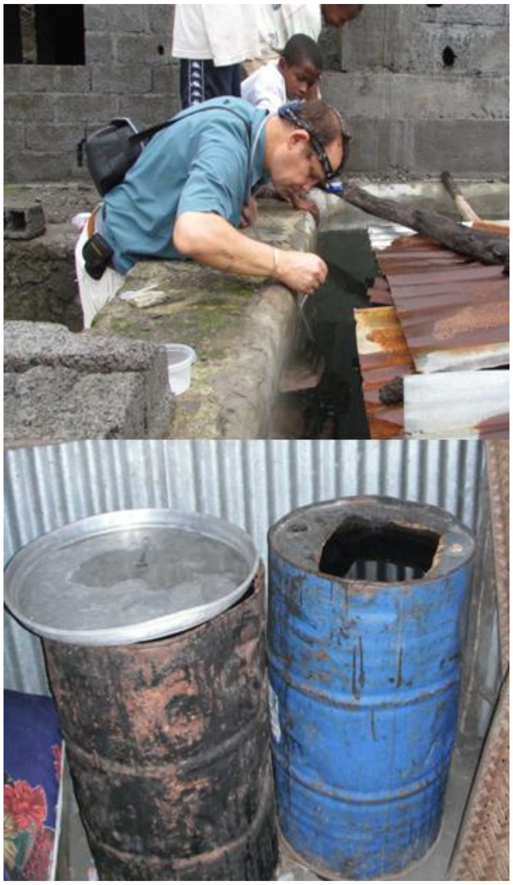

Figure S3: Pairwise Fst values for sites within islands (above diagonal) and geographic distances (below the diagonal). Significan values are shown in bold. Site codes correspond with Table 1.

Grande Comore

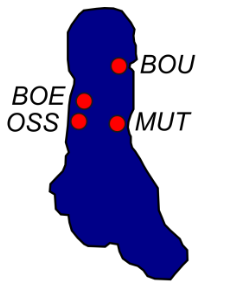

	**BOE**	**BOU**	**MUT**	**OSS**
**BOE**		**0.044**	0.031	0.008
**BOU**	**15 km**		**0.059**	**0.071**
**MUT**	12 km	**13 km**		0.052
**OSS**	3 km	**17 km**	13 km	

Moheli

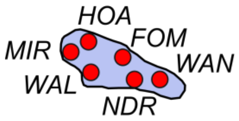

	**HOA**	**FOM**	**WAN**	**MIR**	**WAL**	**NDR**
**HOA**		0.000	0.010	0.008	0.002	0.010
**FOM**	7 km		0.000	0.009	0.000	0.000
**WAN**	17 km	11 km		0.013	0.000	0.000
**MIR**	6 km	11 km	18 km		0.000	0.002
**WAL**	9 km	10 km	14 km	5 km		0.000
**NDR**	14 km	9 km	5 km	9 km	9 km	

Figure S4: Mitochondrial ND5 haplotype network based on trimmed length (665 bp) sequences detected in this study combined with published sequences from East Africa (Besansky et al. 1997; Lehmann et al. 1997; Donnelly, Licht, and Lehmann 2001; Donnelly et al. 2004). Circles represent haplotypes and are coloured according to sampling location, and sized proportional to haplotype frequency. Black nodes denote unsampled haplotypes. Haplotype codes are explained in Table S10.

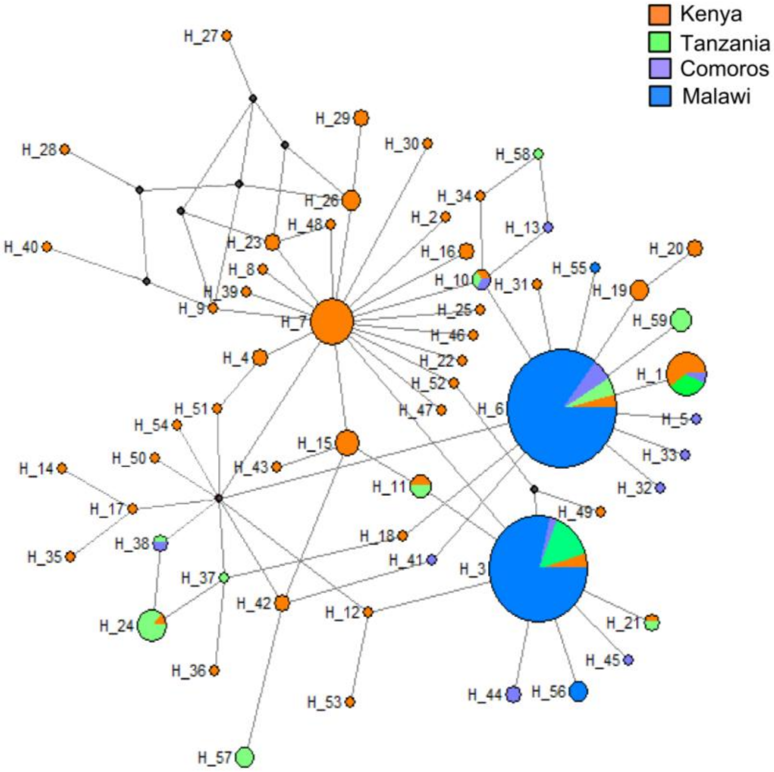

Figure S5 Elevation map for Grande Comore. Boxed area depicts the southern region which is potentially isolated due to topography.

Figure adapted from Wikimedia Commons File: Grande_Comore_topographic_map-fr" by Ikonact.

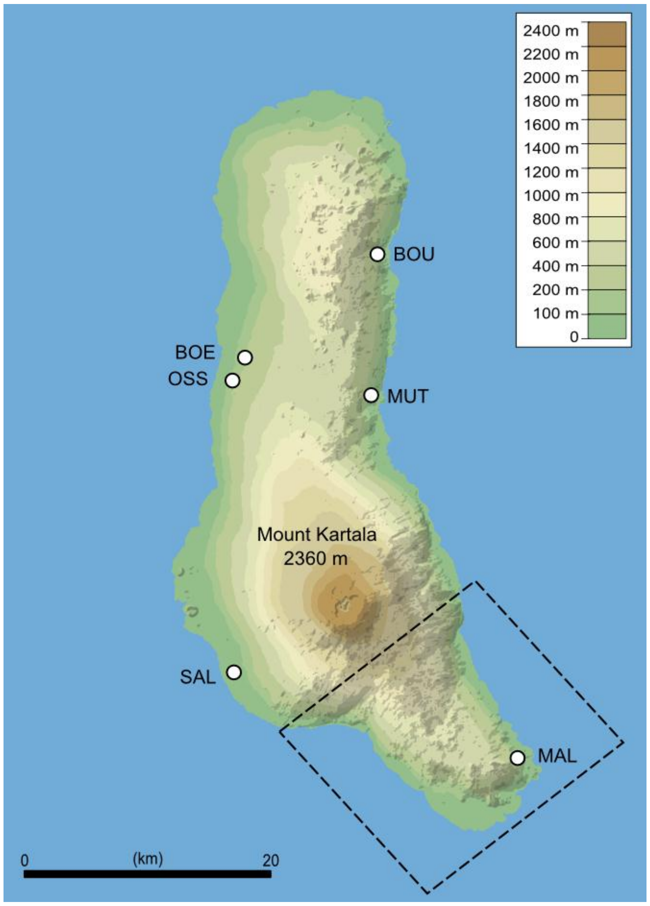

<u>Anecdotal notes on mosquito species collected in Comoros</u>

Table S1. Location and habitat characteristics of *Anopheles* on the Bijagós archipelago.

Village and town Location	*Anopheles gambiae s.l*	*Anopheles rufipes rufipes*	*Anopheles funestus*	*Anopheles pharoensis*	Notes
**Mainland Guinea Bissau**					
Antula	x	x	x	x	Resting indoors
Prabis	x	x	x	x	Resting indoors
**Formosa**					
Abu	x				Resting indoors
**Bubaque**					
Bubaque	x				Larvae in roadside pool at hospital entrance, no vegetation
Brus	x				Roadside pool, vegetation on three sides
**Orango**					
Orango	x				Flying on hotel patio at sunset (sweep net collection)
Eticoga	x				Swamp

Table S2: Location and habitat characteristics of *Aedes* and other genera on the Bijagós archipelago.

Village and town Location	*Aedes aegypti*	*Aedes luteocephalus*	*Aedes mixtus*	*Aedes vittatus*	*Mansonia africana*	*Mansonia uniformis*	Notes
**Mainland Guinea Bissau**							
Antula			x				Resting indoors
Prabis					x	x	Resting indoors
**Formosa**							
Abu	x						Resting indoors
**Bubaque**							
Bubaque				x			Larvae in roadside pool at hospital entrance, no vegetation
**Orango**							
Orango	x	x					Flying on hotel patio at sunset (sweep net collection)
Orango		x					Pot plants holding water

Table S3: Location and habitat characteristics of *Culex* and other genera on the Bijagós archipelago.

Village and town Location	*Culex antennatus*	*Culex decens*	*Culex invidiousus*	*Culex neavei*	*Culex nebulosus*	*Culex quinquefasciatus*	*Culex rima*	*Culex wiggelsworthi*	*Eretmapodites quinquevittatus*	*Coquillettidia aurites*	Notes
**Mainland Guinea Bissau**											
Antula		x			x	x	x	x			Resting indoors
Prabis		x		x	x	x					Resting indoors
**Formosa**											
Abu											Resting indoors
**Bubaque**											
Bubaque		x	x								Larvae in roadside pool at hospital entrance, no vegetation
Bubaque					x	x			x	x	Resting in tall grass under trees
Bubaque	x										Small container in the sun filled with water
Brus					x						Resting indoors

Table S4: Location and habitat characteristics of *Aedes* mosquitoes collected in Comoros islands.

Village and town Location	*Aedes aegypti*	*Aedes albopictus*	*Aedes bromeliae*	*Aedes m,onettus*	*Aedes fowleri*	*Aedes vittatus*	*Aedes cartroni*	Notes
**Grande Comore**								
Moroni	X		X[1]					Water bottle nestled in between leaves of large agave
Moroni	X		X					Coconut shell
Moroni			X					Leaf axils of a variegated colored cotyledon.
Moroni	X		X					Pineapple plant leaf axils
Boenindi	X							Concrete cistern
Ossivo						X		Concrete cistern
Half way between Moroni and Boenindi					X			Vernal pool with short grass and sedges and red soil substrate
Mutsamudu						X		Concrete floor of partially built house
Malé					X	X		Mountain stream with filamentous algae, rocks and sand
Moroni					X	X		Pools of water in mountain stream, rocky substrate and sandy leave filled pools as well
Moroni	X							Larvae in concrete building block filled with water
Moroni	X		X			X		Flying around ankles in hotel gardens
**Anjouan**								
Mutsmudu	X	X[2]	X	X				Sweep netting through vegetation in grounds of hotel during the day
Assimpao							X	Polluted river mouth, some vegetation and rubbish
Mutsamudu						X		Tire
Domino						X		Muddy roadside pool, no vegetation
North of Hohoja	X							Tree hole- Ylang Ylang plantation
South of Hohoja						X		Large plastic container
**Moheli**								
Wanani						X		Muddy shallow pool on edge of soccer field
Ndremeani					X	X		Roadside pool, no vegetation
Wala							X	River mouth, might be brackish, very muddy
Miringoni							X	Slow moving mountain stream pools, village huts next to stream
Fomboni			X					Flying around on patio of hotel

[1] Careful examination of mosquitoes collected resembling members of the *Aedes simpsoni* complex revealed that all had leg banding patterns within the range established by Huang (1979) to be that of *Aedes (Steg.) bromeliae* Theobald.

[2] Several female and males of *Aedes (Steg.) albopictus* were collected resting in the vegetation of Hotel Al Amal in the town of Mutsamuda (Anjouan). *Aedes albopictus* were first recorded as having invaded the Comoros Island archipelago by Girod (2004) on Mayotte Island in 2001. Anjouan is the closest of the three islands we assessed to Mayotte from which this mosquitoes likely invaded and established since 2001. This is the first record of this species which is a vector of arboviruses including Dengue fever (Calisher et al. 1981) and Chikungunya (Delatte et al. 2008) on the island of Anjouan.

Table S5: Location and habitat characteristics of *Culex* mosquitoes collected in Comoros islands.

Location	*Culex comorensis*	*Culex decens*	*Culex simpsoni*	*Culex ethiopicus*	*Culex sunyanensis* like	*Culex quinquefasciatus*	*Culex* unknown	Notes
**Grande Comore**								
Moroni			X			X		Water bottle caught in between leaves of large agave
Boenindi			X					Concrete cistern
Boenindi						X		50 gallon drum indoors
Ossivo			X			X		Concrete cistern
Half way between Moroni and Boenindi			X					Vernal pool with short grass and sedges and red soil substrate
Bouni						X		Concrete cistern
Mutsamudo		X	X			X		Broken Cistern with grass and sedge on edges
Mutsamudu			X			X		Concrete floor of partially built house
Malé		X						Mountain stream with filamentous algae, rocks and sand
Moroni	X	X				X		Pools of water in mountain stream, rocky substrate and sandy leave filled pools as well
**Anjouan**								
Assimpao			X		X[1]	X		Polluted river mouth, some vegetation and rubbish
2km north of Assimpao		X	X					Roadside ditch with short grassy vegetation all around
Simo	X	X	X				X	Mountain stream, muddy in appearance, steep embankment, no vegetation
Chandrè	x	x						Mountain stream pool, small sandy bottom
Tsembehou	X							Mountain stream pool, large rocky and sandy substrates
Outskirts of town of Mutsamudu						X		Tire
Bambao-Mtsanga			X	X				Marshy area with vegetation created by leaking aquifer
Moya			X					Shady mountain stream pool with rocky and sandy substrate some submerged vegetation
Moya			X					Swampy vernal pool, large, much submerged vegetation and detritus
Sadapoini		X	X					Mountain stream pool, muddy water
**Moheli**								
Wala			X					River mouth, might be brackish, very muddy
Fomboni			X					Muddy pools in town.
Miringoni			X			X		Slow moving mountain stream pools, village huts next to stream
A few kilometers north of Miringoni	X							Small deep mountain stream
Fomboni						X		Flying around ankles on Hotel patio

[1] A single larva collected at Assimpao was reared to an adult male. The larval pelt characters resembled *Culex (Eum.) sunyaniensis* Edwards in all aspects. These included presence of an unpaired mid ventral tuft proximal to the multi-branched paired tufts in the barred area, subventral tufts shorter than width of siphon and non spiculated distal margin of saddle. All these characters distinguish this species from larvae of closely related *Culex (Eum.) wigglesworthi* Edwards which has subventral tufts longer than the width of the siphon, speculated saddle margin and three small unpaired ventral tufts outside barred area (Hopkins 1952). Adult male however, keyed out as *Cx. wigglesworthi* because of a large patch of white erect head scales (Edwards 1941) and shape and numbers of appendages on basal lobe of male coxite (Hamon and Rickenbach 1955). This is the first record of either of these species occurring in the Comoro Islands.

Table S6: Location and habitat characteristics of *Anopheles* mosquitoes collected in Comoros islands.

Location	*Anopheles gambiae*	*Anopheles pretoriensis*	*Anopheles coustani*	*Anopheles* unknown	Notes
**Grande Comore**					
Boenindi	X				Concrete cistern
Boenindi	X				50 gallon drum indoors
Ossivo	X	X			Concrete cistern
Bouni	X				Concrete cistern
Mutsamudu	X	X			Concrete floor of partially built house
Malé	X				Large water filled depression between village houses
Malé	X	X			Mountain stream with filamentous algae, rocks and sand
**Anjouan**					
Assimpao		X[1]			Polluted river mouth, some vegetation and rubbish
Assimpao	X	X			Roadside ditch with short grassy vegetation all around
Tsembehou		X			Mountain stream pool, large rocky and sandy substrates
Bambao-Mtsanga		X		X[2]	Marshy area with vegetation created by leaking aquifer
Moya		X			Shady mountain stream pool with rocky and sandy substrate some submerged vegetation
Moya	X				Swampy vernal pool, large, much submerged vegetation and detritus
Sadapoini		X			Mountain stream pool, muddy water
Hohoja		X			Mountain stream pool near river mouth, sandy and rocky substrates
**Moheli**					
Wanani	X				Muddy shallow pool on edge of soccer field
Ndremeani	X	X			Roadside pool, no vegetation
Wala	X				River mouth, might be brackish, very muddy
Fomboni	X				Muddy pools in town.
Hoani	X	X			Roadside pools, muddy, no vegetation
Miringoni	X	X	X		Slow moving mountain stream pools, village huts next to stream

[1] Wing pale markings of *Anopheles pretoriensis* originating from all three islands consistently varied slightly from that described by Gillies and DeMeillon (1968) (Figure S1).

[2] Unknown Anopheles species. A single female reared from a pupa collected in the swamp could be keyed out in Gillies and DeMeillon (1968) or in Gillies and Coetzee (1987). The specimen is a small *Anopheles* that does not key out beyond couplet 7 (section IX) in Gillies and Coetzee (1987) because of the apical pale banding patterns that that are very small and indistinct on mid and hind tarsal segments 1-3 (absent in 4 and 5) and more distinct and broader on tarsal segments 1-3 in the front legs. Wing banding also differs from other mosquitoes in section IX that contains mosquitoes closely resembling the *marshalli* group of mosquitoes.

Table S7: Location and habitat characteristics of *Eretmapodites* and other mosquitoes collected in Comoros islands.

Location	*Eretmapodites quinquevittatus*	*Eretmapodites subsimplicipes*	*Lutzia tigripes*	Notes
**Grande Comore**				
Moroni, La Moroni Hotel grounds	X	X		Coconut shell
Ossivo			X	Concrete cistern
Bouni			X	Concrete cistern
Mutsamudu			X	Broken Cistern with grass and sedge on edges
Mutsamudu			X	Concrete floor of partially built house
Hotel Morini	X	X		Flying around ankles in hotel gardens
**Anjouan**				
Hotel Al Amal grounds, Town of Mutsmudu	X	X		Man biting in grounds of hotel during the day
Moya			X	Swampy vernal pool, large, much submerged vegetation and detritus
South of Hohoja			X	Large plastic container
**Moheli**				
Fomboni	X	X		Flying around ankles on Hotel patio

Table S8: Locus specific pairwise F_{ST} values for Tanzania-Comoros island comparisons, with significant values after Bonferoni correction underlined. SNP names reflect ensemble ID's, and are organized according their chromosomal location (2L=left arm of chromosome 2, 2R=right arm of chromosome 2 etc). SNPs located within inversions are denoted with superscripts, [b]=2Rb, [u]=2Ru, [a]=2La.

	TNZ-AJ	TNZ-GC	TNZ-MH
**Ag2L-02422654**	0.96437	0.97394	0.97894
**Ag2L-02422079**	0	0.0791	0
**Ag2L-01272330**	0.01511	0	0.03391
**Ag2L-37733999**[a]	0.26942	0.21809	0.25659
**Ag2L-39892883**[a]	0.16241	0.16994	0.16537
**Ag2L-39996745**[a]	0	0.00029	0.17209
**Ag2L-40902105**[a]	0.01511	0.02532	0.03391
**Ag2R-04012275**	0.31338	0.35015	0.44361
**Ag2R-25147838**[b]	0.06277	0.08849	0.11057
**Ag2R-27040679**	0.32681	0.00244	0.00755
**Ag2R-32389630**[u]	0.01888	0.15585	0
**Ag2R-42820496**	0.03903	0.01914	0.03154
**Ag2R-50098579**	0.13382	0.13378	0.05818
**Ag2R-56681764**	0.03903	0.05729	0.07301
**Ag3L-00125286**	0.21352	0.00281	0.10022
**Ag3L-00387649**	0	0	0
**Ag3L-00670814**	0	0.05523	0.13413
**Ag3L-01242464**	0.0163	0	0.03984
**Ag3L-03093406**	0.20943	0.11915	0
**Ag3L-06551099**	0.18865	0.00233	0.03686
**Ag3L-09844638**	0	0.0749	0.04889
**Ag3L-14050318**	0.09808	0.13391	0.16423
**Ag3L-41786408**	0.00309	0.09499	0.01375
**Ag3R-13897048**	0.27944	0.10957	0.0187
**Ag3R-14846418**	0.00574	0.192	0
**Ag3R-28746909**	0.08295	0.42492	0.20182
**Ag3R-38153793**	0.18769	0.10058	0
**Ag3R-48194942**	0.05464	0.03687	0.02455
**AgX-18983531**	0.00486	0	0
**AgX-21772730**	0.00064	0.40107	0
**AgX-22817756**	0.03903	0.05729	0.07301

Table S9: Frequency of full length ND5 haplotypes by sampling location. Three letter codes correspond with Table 1 and Figure 2.

		Haplotype Frequency					
		Mainland	Comoros islands				
Accession	Haplotype	Tanzania	Grande Comore		Anjouan	Moheli	
		DAR	BOE	BOU	MOY	WAL	WAN
KC249505	ISL01		21	6			
KC249506	ISL02	4	10	25	23		
KC249507	ISL03				6	26	28
KC249508	ISL04				1		
KC249509	ISL05					2	
KC249510	ISL06					1	2
KC249511	ISL07	3					
KC249512	ISL08	3					
KC249513	ISL09	6					
KC249514	ISL10	1					
KC249515	ISL11	4					
KC249516	ISL12	10					
KC249517	ISL13	1					

Table S10: Haplotype numbers from Figure S4 and their corresponding haplotype designations by Besansky and Genbank accession numbers.

Figure S4 H#	H# from Besansky	Genbank accession #
1	1	AF020965*
*	2	AF020966*
2	3	AF020967
3	11	AF020975
4	14	AF020978
5	31	AF020995
6	32	AF020996
7	33	AF020997
8	35	AF020999
9	36	AF021000
10	37	AF021001
11	41	AF021005
12	42	AF021006
13	43	AF021007
14	47	AF021011
15	48	AF021012
16	49	AF021013
17	50	AF021014
18	51	AF021015
19	52	AF021016
20	53	AF021017
21	54	AF021018
22	55	AF021019
23	56	AF021020
24	57	AF021021
25	58	AF021022
26	59	AF021023
27	62	AY312092
28	63	AY312093
29	65	AY312094
30	67	AY312096
31	68	AY312097
32	75	AY312104
33	76	AY312105
34	78	AY312107
35	80	AY312109
36	81	AY312110
37	82	AY312111

38	83	AY312112
39	87	AY312116
40	90	AY312119
41	93	AY312122
42	94	AY312123
43	95	AY312124
44	98	AY312127
45	100	AY312129
46	102	AY312131
47	103	AY312132
48	107	AY312136
49	108	AY312137
50	109	AY312138
51	110	AY312139
52	111	AY312140
53	112	AY312141
54	113	AY312142
55	ISL04-trimmed	TableS9
56	ISL06-trimmed	TableS9
57	ISL08-trimmed	TableS9
58	ISL10-trimmed	TableS9
59	ISL11-trimmed	TableS9

* AF020965 & AF020966 sequences uploaded to GENBANK were identical.

References

Besansky, N., T. Lehmann, G. Fahey, D. Fontenille, L. Braack, W. Hawley, and F. Collins. 1997. Patterns of mitochondrial variation within and between African malaria vectors, *Anopheles gambiae* and *An. arabiensis*, suggest extensive gene flow. Genetics **147**:1817-1828.
Calisher, C. H. Nuit, M., Lazuick J.S., Ferrari, J. D. M., and Kappus, K. D. Dengue in the Seychelles. Bull. World Hlth. Org. 1981. 59: 619-622.
Delatte H., Paupy, C., Dehecq J. S., Thiria, J., Failloux, A. B., Fontenille, D. 2008. Aedes albopictus, vector of chikungunya and dengue viruses in Reunion Isalnd :biology and control. Parasite 15: 3-13.
Donnelly, M., M. Licht, and T. Lehmann. 2001. Evidence for recent population expansion in the evolutionary history of the malaria vectors *Anopheles arabiensis* and *Anopheles gambiae*. Molecular Biology and Evolution **18**:1353-1364.
Donnelly, M. J., J. Pinto, R. Girod, N. J. Besansky, and T. Lehmann. 2004. Revisiting the role of introgression vs shared ancestral polymorphisms as key processes shaping genetic diversity in the recently separated sibling species of the *Anopheles gambiae* complex. Heredity **92**:61-68.
Edwards F. W. 1941. Mosquitoes of the Ethiopian Region III. Culicine adults and pupae. British Museum of Natural History, London.
Gillies M. T and DeMeillon B. 1968. The anophelinae of Africa south of the Sahara. The South African Institute for Medical Research, Johannesburg.
Gillies M. T and Coetzee, M., 1987. The South African Institute for Medical Research, Johannesburg.
Girod R. 2004. First record of *Aedes albopictus* in Mayotte Island, Comoros Archipelago. Parasite 11: 74
Hamon J and Rickenbach, A. 1955. Contribution a L'étude des Néoculex (Diptéres: Culicidés) de la Région Éthiopienne. 1. Corrections de quelques descriptions de terminalia mâles données par Edwards, avec étude d'une nouvelle variété. Bull. Soc. De Pathologie exotique. 48 :6 ; 848-859.
Hopkins G. H. E. 1952. Mosquitoes of the Ethiopian region I. Larval bionomics of mosquitoes and taxonomy of Culicine larvae. British Museum of Natural History, London.
Huan, Y. M. 1979. *Aedes (stegomyia) simpsoni* complex in the Ethiopian region with lectotype designation for *simpsoni* (Theobald) (Diptera:Culicidae). Mosquito Systematics 11: 221-234.
Lehmann, T., N. Besansky, W. Hawley, T. Fahey, L. Kamau, and F. Collins. 1997. Microgeographic structure of *Anopheles gambiae* in western Kenya based on mtDNA and microsatellite loci. Molecular Ecology **6**:243-253.
